# Zanamivir aqueous solution in severe influenza: A global Compassionate Use Program, 2009–2019

**DOI:** 10.1111/irv.12947

**Published:** 2021-12-22

**Authors:** Jie Wang‐Jairaj, Irene Miller, Aditya Joshi, Tharaka Jayabalan, Amanda Peppercorn, Peter Zammit‐Tabona, Amanda Oliver

**Affiliations:** ^1^ Clinical Science, R&D GSK Brentford Middlesex UK; ^2^ Safety and Medical Governance, R&D Global Medical GSK Brentford Middlesex UK; ^3^ Development Biostatistics GSK Bangalore India; ^4^ Clinical Development GSK Boston Massachusetts USA; ^5^ Global Clinical Science and Delivery, R&D GSK Collegeville Pennsylvania USA; ^6^ Global Health R&D GSK Brentford Middlesex UK; ^7^ Present address: Galecto Biotech AB London UK

**Keywords:** compassionate use, hospitalized, human influenza, intravenous zanamivir, safety

## Abstract

**Background:**

Zanamivir is a neuraminidase inhibitor effective against influenza A and B viruses. In 2009, GlaxoSmithKline (GSK) began clinical development of intravenous (IV) zanamivir and initiated a global Compassionate Use Program (CUP) in response to the evolving H1N1 global pandemic. The goal of the CUP was to provide zanamivir to critically ill patients with limited treatment options.

**Methods:**

Zanamivir was administered to patients with suspected or confirmed influenza infection who were not suitable for other approved antiviral treatments. Reporting of serious adverse events (SAEs) was mandatory and recorded in the GSK safety database. A master summary tracking sheet captured requests and patient characteristics. A case report form was available for detailing medical conditions, dosing, treatment duration, and clinical outcomes.

**Results:**

In total, 4,033 requests were made for zanamivir treatment of hospitalized patients from 38 countries between 2009 and 2019; ≥95% patients received zanamivir via the IV route. Europe had the highest number of requests (*n* = 3,051) followed by North America (*n* = 713). At least 20 patients were aged ≤6 months, of whom 12 were born prematurely. The GSK safety database included 466 patients with ≥1 SAE, of whom 374 (80%) had a fatal outcome. Drug‐related SAEs were reported in 41 (11%) patients, including hepatic failure (*n* = 6 [2%]) and acute kidney injury (*n* = 5 [1%)].

**Conclusions:**

The CUP facilitated global access to zanamivir prior to product approval. No new safety concerns were identified in the CUP compared with IV zanamivir clinical studies.

## 1 INTRODUCTION

Seasonal influenza, an acute respiratory infection, leads to 3–5 million cases of severe illness and 290,000–650,000 respiratory deaths globally each year.^1^ Influenza pandemics occur in waves, adding to the global burden. Adamantanes and neuraminidase inhibitors are two classes of antiviral medicines approved in most countries.^2^, ^3^ Baloxavir marboxil, a selective inhibitor of influenza cap‐dependent endonuclease, is approved in the United States, EU, and Japan.^4^, ^5^ Neuraminidase inhibitors (oseltamivir, zanamivir, and peramivir) have antiviral activity against all influenza A and B subtypes and are effective in the treatment and prophylaxis of influenza.^6^, ^7^ Compared with oseltamivir and peramivir, zanamivir displays efficacy against the most common influenza A virus resistance mutation, H275Y.^8^, ^9^ In 2009, following the emergence of the influenza A/H1N1pmd09 global pandemic, GSK initiated a clinical development program for an aqueous formulation of zanamivir for intravenous (IV) administration at the request of the Food and Drug Administration (FDA) in the United States. In parallel, a global Compassionate Use Program (CUP) supplied zanamivir aqueous solution through an Emergency Investigational New Drug or named‐patient process in the United States and local regulations including under Article 83 in parts of Europe, for IV or nebulized use to seriously ill patients with influenza infection for whom approved anti‐influenza drugs were not effective or feasible.

Zanamivir powder for inhalation (RELENZA, zanamivir, GSK, United Kingdom) has been approved for >20 years for the treatment and prophylaxis of influenza A and B in adults and children ≥5 years of age in over 50 countries, including the EU, Japan, and the United States.^10^, ^11^ The clinical development program investigated IV zanamivir in adult and pediatric patients (between 6 months and 18 years of age) hospitalized with influenza.^12^, ^13^, ^14^ In 2019, GSK received marketing authorization for IV zanamivir in Europe for the treatment of complicated and potentially life‐threatening influenza A or B virus infection in adult and pediatric (≥6 months of age) patients for influenza virus known or suspected to be resistant to anti‐influenza medicinal products other than zanamivir, and/or when other anti‐viral medicinal products for treatment of influenza, including inhaled zanamivir, are not suitable for the individual patient.^10^ The CUP was terminated following this marketing authorization.

Patient characteristics, zanamivir dosing information and safety data, including serious adverse events (SAEs), were collected from 2009 to 2019. This current report presents analysis of all available CUP data on zanamivir aqueous solution received up to January 31, 2020.

## 2 METHODS

### 2.1 CUP design and initiation

The CUP was administered via applicable country regulatory requirements, with the requesting physician considered the designated sponsor. A Physician Guidance Document was developed to support requests for IV and nebulized zanamivir on a compassionate use named‐patient basis and provide guidance on dosing, administration, and patient eligibility. Similarly, the European Medicines Agency published a “Condition of Use” document to give guidance for IV zanamivir requests via Article 83. The CUP was referenced through governmental information sources, including public health influenza advice websites.^15^ The CUP process is summarized in Figure S1 and in Methods S1.

### 2.2 Dosing and administration

Recommendations for IV administration of zanamivir specified in the “Guidance for physicians” was 600 mg in a 30‐min infusion, every 12 h, adjusted for age, weight (for pediatric patients), renal function, and renal replacement modality. Nebulized zanamivir was recommended at a dose of 25 mg, four times/day. The recommended duration of initial treatment was 5 days (Methods S1).

### 2.3 Eligibility criteria

Patients were eligible if, hospitalized and severely ill with influenza infection and not responding to authorized antiviral medicinal products (e.g., oral oseltamivir or inhaled zanamivir), or for whom drug delivery by a route other than IV was not expected to be dependable or feasible, or if there was resistance to other antiviral agents and inhaled zanamivir was not suitable.^16^

### 2.4 Data collection

Baseline and follow‐up data were summarized across three databases (Table 1). The master summary tracking sheet (MSTS) provided information on patient demographics and overall medical condition, with a data cut‐off of May 6, 2019. All SAEs were required to be reported to local ethics committees and regulatory authorities per regulatory agency requirements and were recorded in the GSK safety database. Data cut‐off was extended to January 31, 2020, (beyond termination of IV zanamivir supplies in the CUP) to allow adequate time for additional safety reports and/or follow‐up information.

**TABLE 1:** Patient demographic characteristics by data source (MSTS, CRF, and GSK safety database)

	MSTS *N* = 4033	CRF *N* = 877	GSK safety database *N* = 466
Age^a^	*n* = 4014	*n* = 817	*n* = 422
Mean (SE)	47.3 (0.32)	44.5 (0.70)	44.3 (0.96)
Min–max.	0–98	0–100	0–90
Age category^a^, *n* (%)	—	—	—
0 to <6 months	9 (<1)	9 (1)	5 (1)
0 to ≤6 months^b^	20 (<1)	—	—
6 months to ≤1 year	82 (2)	15 (2)	4 (<1)
>1 to ≤2 years	42 (1)	7 (<1)	7 (2)
>2 to ≤5 years	84 (2)	28 (3)	13 (3)
>5 to ≤12 years	105 (3)	21 (2)	12 (3)
>12 to ≤17 years	84 (2)	21 (2)	13 (3)
18 to ≤64 years	2860 (71)	600 (68)	312 (67)
65 to ≤74 years	480 (12)	82 (9)	41 (9)
75 to ≤84 years	206 (5)	32 (4)	13 (3)
85 to ≤94 years	57 (1)	1 (<1)	2 (<1)
≥95 years	5 (<1)	1 (<1)	0
Missing/unknown	19 (<1)	62 (7)	44 (9)
Sex, *n* (%)	*n* = 4033	*n* = 879	*n* = 466
Female	1653 (41)	355 (40)	205 (44)
Male	2335 (58)	498 (57)	251 (54)
Missing/unknown	45 (1)	26 (3)	10 (2)
Pregnancy, *n* (%)	60 (4)	16 (5)	9 (4)
Ethnicity, *n* (%)	—	—	—
Hispanic or Latino	—	56 (6)	—
Not Hispanic or Latino	—	357 (41)	—
Missing	—	466 (53)	—
Geographic ancestry, *n* (%)	—	n = 879	—
African American/African heritage	—	35 (4)	—
American Indian or Alaskan native	—	5 (<1)	—
Asian—Central/South Asian	—	7 (<1)	—
Asian—East Asian Heritage	—	9 (1)	—
Asian—South East Asian Heritage	—	19 (2)	—
White—Arabic/North African Heritage	—	6 (<1)	—
White—White/Caucasian/European Heritage	—	302 (34)	—
Missing	—	496 (56)	—

Abbreviations: CRF, case report form; GSK, GlaxoSmithKline; MSTS, master summary tracking sheet; SE, standard error.

^a^ Due to incomplete date of birth information for some patients, it was not feasible to calculate age. Calculated age across data sets may differ by 1–2 years depending on when and how age was computed.

^b^ Standalone analysis of infants and neonates aged ≤6 months.

A case report form (CRF) was provided to collect information on medical conditions, zanamivir dosing, treatment duration, and clinical outcomes (data cut‐off: January 31, 2020). Completion and return of this form to the sponsor was encouraged but not mandatory for supply of aqueous zanamivir. Modifications made to the CRF template over the CUP minimized the collection of unusable data (Table S1).

The information collected in each data source is summarized in Table S2, with additional information regarding the databases in Methods S1.

### 2.5 AE reporting

Adverse events (AEs) meeting the definition of a SAE in patients who received ≥1 dose of zanamivir from the time of the first dose until 14 days after treatment completion were required to be reported. Any SAEs reported after this time were included in the GSK safety data set. SAEs were summarized separately for the pediatric population. Further description of AE reporting is provided in Methods S1.

### 2.6 Statistical methods

Data from the three databases (MSTS, CRF, and GSK safety) were summarized depending on data type. Frequency and percentages were reported for categorical outcomes such as AEs, SAEs, and chronic underlying illness. Mean (standard error) was reported for continuous outcomes such as age and ventilation data. A separate examination of the MSTS was performed to summarize all available information about infants and neonates aged ≤6 months.

### 2.7 Role of the funding source

GSK contributed to the design and collection, analysis, and interpretation of data. Authors employed by GSK participated in the writing, review, and approval of the manuscript. All authors had access to the data and approved the final manuscript for submission.

## 3 RESULTS

### 3.1 CUP global enrollment and completion of CRF

At data cut‐off (May 6, 2019), requests for zanamivir treatment were received for 4,033 patients globally, with most requests from Europe (*n* = 3,051) followed by North America (*n* = 713) (Table 2). Treating physicians returned CRFs for 877 patients. The highest CRF completion rates were in Europe (*n* = 428) and North America (*n* = 379), which included 184 and 356 patients from the United Kingdom and the United States, respectively.

**TABLE 2:** Summary of requests for CUP enrollment by continent and country from MSTS and CRFs

	MSTS, *n*	CRFs, *n*
Asia	136	29
China	5	0
Hong Kong	95	14
Korea	1	0
Singapore	27	11
Thailand	8	4
Australia	103	32
Europe	3,051	428
Austria	12	2
Belgium	7	0
Cyprus	7	1
Denmark	67	14
Estonia	2	2
France	135	26
Germany	253	81
Gibraltar	1	1
Greece	184	30
Ireland	72	7
Italy	35	12
Latvia	5	5
Lithuania	2	1
Netherlands	63	5
Norway	17	2
Poland	1	0
Portugal	38	9
Romania	1	0
Slovakia	1	0
Spain	121	13
Sweden	1	0
Switzerland	76	33
United Kingdom	1,950	184
Middle East	27	9
Bahrain	1	0
Israel	16	6
Oman	1	1
Saudi Arabia	1	0
United Arab Emirates	8	2
North America	713	379
Canada	81	23
United States	632	356
South America	3	0
Argentina	1	0
Brazil	2	0

Abbreviations: CRF, case report form; CUP, Compassionate Use Program; MSTS, master summary tracking sheet.

### 3.2 Baseline characteristics

**FIGURE 1:**
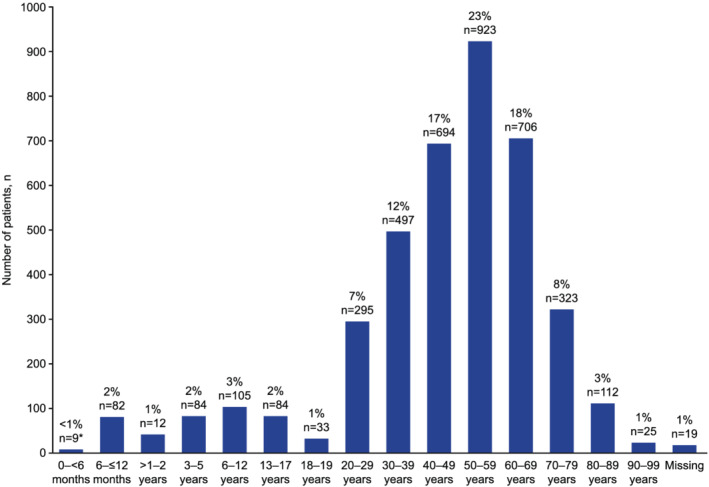
Age distribution for patients enrolled on CUP from the MSTS. *Does not include standalone analysis of infants and neonates aged ≤6 months. CUP, Compassionate Use Program; MSTS, master summary tracking sheet

The 4,033 patients included in the CUP based on MSTS data had a mean age of 47.3 years (range: 0–98) (Table 1); over 50% were aged 40–69 years (Figure 1), with a slightly higher proportion of males (2,335 [58%] patients). Among 1,653 female patients, 60 (4%) were pregnant at the time of zanamivir request (Table 1). Based on the standalone evaluation of the MSTS for infants and neonates, at least 20 patients were aged ≤6 months, of whom 12 were born prematurely with a gestational age of 23–35 weeks.^17^ The 877 patients (including 16 pregnant women) in the CRF database had a mean age of 44.5 years, with more than three quarters (*n* = 681 [77%]) having chronic underlying illnesses or risk factors, the most common being respiratory illness recorded in 320 (36%) patients (Table 3).

**TABLE 3:** Summary of chronic underlying illness and risk factors in ≥1% of patients receiving zanamivir on the CUP registered in the CRF database

Chronic underlying illness/risk factor, *n* (%)	CRF (*N* = 877)
Any illness or risk factor	681 (77)
Respiratory	320 (36)
Tobacco use	190 (22)
Chronic obstructive pulmonary disease	76 (9)
Asthma	71 (8)
Chronic lung disease	54 (6)
Chronic supplemental oxygen	15 (2)
Rheumatology and immunology	295 (34)
Any immunocompromise^a^	168 (19)
Leukemia/lymphoma	129 (15)
Organ/bone marrow transplantation	76 (9)
Rheumatoid arthritis	19 (2)
HIV/AIDS	16 (2)
Vasculitis	14 (2)
Gastrointestinal disease	155 (18)
Morbid obesity	96 (11)
Malnutrition	27 (3)
Cirrhosis/chronic liver disease	23 (3)
Crohn's disease/inflammatory bowel disease	14 (2)
Endocrine disease	137 (16)
Diabetes mellitus	132 (15)
Oncology	128 (15)
Current cancer or cancer treatment with 1 year	128 (15)
Cardiovascular	126 (14)
Coronary artery disease	67 (8)
Arrythmia	40 (5)
Congestive heart failure	31 (4)
Cardiomyopathy	20 (2)
Renal disease	78 (9)
Chronic renal insufficiency	64 (7)
End stage renal disease: hemodialysis	17 (2)
Neurology	43 (5)
Seizure disorder	22 (3)
Stroke/cerebral vascular disease	17 (2)
Newborn prematurity	10 (1)

Abbreviations: AIDS, acquired immune deficiency syndrome; CRF, case report form; CUP, Compassionate Use Program; HIV, human immunodeficiency virus.

^a^ Including use of immunosuppressive medication.

### 3.3 Zanamivir formulation, dosages, and dosing duration

A summary of zanamivir treatment captured in the MSTS and CRF databases is provided in Table 4. The majority (≥95%) of patients received zanamivir via the IV route. Based on MSTS data, an additional treatment course (beyond the initial 5‐day course) was requested for nearly 25% of patients (range: 1–75 days).

**TABLE 4:** Summary of zanamivir treatment for all patients registered to the MSTS and CRF databases

	MSTS (*N* = 4,033)	CRF (*N* = 877)
Route of administration, *n* (%)	*n* = 4,033	*n* = 877
IV	3,879 (96)	823 (94)
Nebulized	144 (4)	33 (4)
Both IV and nebulized	9 (<1)	11 (1)
Missing	1 (<1)	10 (1)
Treatment duration, days	—	*n* = 820
Mean (SE)	—	6.9 (0.17)
Duration of dose, *n* (%)	*n* = 4,033	—
5 days	3,072 (76)	—
˃5 days	961 (24)	—
Additional courses of treatment, *n* (%)^a^	n = 961	—
1 (5‐day initial course + 5 days)	767 (80)	—
2 (5‐day initial course + 10 days)	147 (15)	—
≥3 (5‐day initial course + ≥ 15 days)	47 (5)	—
Dose adjustment due to change in renal function, *n* (%)	—	*n* = 877
Yes	—	231 (26)
No	—	341 (39)
Missing	—	305 (35)
Zanamivir treatment stopped prematurely, *n* (%)	—	*n* = 877
Yes	—	155 (18)
No	—	547 (62)
Missing	—	175 (20)
Reason for stopping zanamivir prematurely, *n* (%)	—	*n* = 155
AE	—	57 (6)
Death	—	3 (<1)
Treating physician's discretion	—	64 (7)
Decision by patient or proxy	—	10 (1)
Other	—	9 (1)
Missing	—	12 (1)

Abbreviations: AE, adverse event; CRF, case report form; IV, intravenous; MSTS, master summary tracking sheet; SE, standard error.

^a^ Number of additional courses in the 961 patients who received >5 days of treatment; treatment supplied does not indicate received; data for patients aged ≥18 years.

### 3.4 Clinical outcomes recorded in the CRFs

Among the 877 patients in the CRF database, 30% (*n* = 267) recovered or their condition resolved, 36% (*n* = 320) had not recovered or their condition was not resolved at the time of CRF completion, 26% (*n* = 231) died, <1% (*n* = 1) were recovering/resolving, and no outcome was recorded for 7% (*n* = 60) patients. Three patients <6 months of age reported SAEs (pneumonitis, cardiopulmonary failure, and lung disorder) with a fatal outcome.

### 3.5 Serious adverse events

Overall, 466 patients reported ≥1 SAE based on data included in the GSK safety database with a total of 839 SAEs recorded, with a higher proportion of males (*n* = 251; 54%) and over 80% (*n* = 384) reporting previous or ongoing medical conditions or interventions (Table S3). Of the 466 patients, 374 (80%) had a fatal outcome (Table 5), and 86 (18%) had at least one drug‐related SAE (in the opinion of the treating/reporting physician). Three drug‐related SAEs were reported in ≥1% of these patients, including acute kidney injury (*n* = 8; 2%) hepatic failure (*n* = 6; 1%), and alanine aminotransferase increased (*n* = 5; 1%). Drug‐related SAEs were reported in 41/374 (11%) patients with a fatal outcome; two drug‐related SAEs were reported in ≥1% of these patients (hepatic failure [*n* = 6; 2%] and acute kidney injury [*n* = 5; 1%]).

**TABLE 5:** SAE recorded in the GSK safety database

SAE^a^ (preferred term), *n* (%)	All patients *N* = 466	All patients with a fatal outcome *N* = 374	Pediatric patients with a fatal outcome *N* = 41
Death^b^	86 (18)	86 (23)	5 (12)
Respiratory failure	59 (13)	54 (14)	11 (27)
Multiple organ dysfunction syndrome	52 (11)	52 (14)	3 (7)
Acute respiratory distress syndrome	46 (10)	44 (12)	5 (12)
Septic shock	27 (6)	26 (7)	2 (5)
Acute kidney injury	25 (5)	17 (5)	—
Cardiac arrest	19 (4)	17 (5)	2 (5)
Pneumonia	18 (4)	18 (5)	1 (2)
Hypoxia	17 (4)	17 (5)	2 (5)
Renal failure	17 (4)	12 (3)	3 (7)
Influenza	16 (3)	15 (4)	2 (5)
Pneumothorax	16 (3)	10 (3)	1 (2)
H1N1 influenza	14 (3)	13 (3)	1 (2)
Hepatic failure	9 (2)	8 (2)	—
Hemorrhage intracranial	8 (2)	7 (2)	2 (5)
Thrombocytopenia	8 (2)	5 (1)	—
Cerebral hemorrhage	7 (2)	6 (2)	1 (2)
Cholestasis	7 (2)	—	1 (2)
Hemodynamic instability	7 (2)	6 (2)	—
Sepsis	7 (2)	6 (2)	—
Shock	7 (2)	7 (2)	—
Alanine aminotransferase increased	6 (1)	—	—
Hepatocellular injury	6 (1)	—	—
Pulmonary embolism	6 (1)	—	—
Pulmonary hemorrhage	6 (1)	6 (2)	3 (7)
Respiratory distress	6 (1)	—	—
Atrial fibrillation	5 (1)	5 (1)	—
Cardiac failure	5 (1)	5 (1)	1 (2)
Gastrointestinal hemorrhage	5 (1)	—	—
Hypotension	5 (1)	—	1 (2)
Brain injury	4 (<1)	—	2 (5)
Cerebrovascular accident	4 (<1)	—	1 (2)
Pneumonia viral	4 (<1)	—	1 (2)
Respiratory disorder	4 (<1)	—	1 (2)
Staphylococcal infection	4 (<1)	—	1 (2)
Cardiopulmonary failure	3 (<1)	—	3 (7)
Disseminated intravascular coagulation	3 (<1)	—	1 (2)
Metabolic acidosis	3 (<1)	—	1 (2)
Pneumonia influenza	3 (<1)	—	1 (2)
Hemophagocytic lymphohistiocytosis	2 (<1)	—	1 (2)
Lung disorder	2 (<1)	—	1 (2)
Anaphylactic shock	1 (<1)	—	1 (2)
Bronchospasm	1 (<1)	—	1 (2)
Cerebral hypoperfusion	1 (<1)	—	1 (2)
Coagulopathy	1 (<1)	—	1 (2)
Hematuria	1 (<1)	—	1 (2)
Hemorrhagic disorder	1 (<1)	—	1 (2)
Intravascular hemolysis	1 (<1)	—	1 (2)
Lower respiratory tract infection	1 (<1)	—	1 (2)
Myocarditis	1 (<1)	—	1 (2)
Nervous system disorder	1 (<1)	—	1 (2)
Oxygen saturation decreased	1 (<1)	—	1 (2)
Pneumonitis	1 (<1)	—	1 (2)
Rash	1 (<1)	—	1 (2)
Serratia sepsis	1 (<1)	—	1 (2)
Transaminases increased	1 (<1)	—	1 (2)

Abbreviation: SAE, serious adverse event.

^a^ A single case can include ≥1 event;

^b^ Reported as “death” but not otherwise specified.

### 3.6 SAEs in patient subgroups

Based on the CRF database, 16/355 female patients (5%) were pregnant, with one, five, and eight patients in the first, second, and third trimester, respectively (two patients had missing data). Information was available for 12 pregnant and four recently postpartum patients in the GSK safety database. Most SAEs reported by these pregnant women were respiratory related; the outcome was fatal in five cases. Outcomes were reported in five of the remaining seven pregnant women: four had a live birth with no apparent congenital anomalies (third trimester exposure, *n* = 3; unknown trimester exposure, *n* = 1); one received a “therapeutic abortion” (first trimester exposure). SAEs were reported in all four postpartum patients, three of which had a fatal outcome (cardiac arrest, multiple organ dysfunction syndrome, and septic shock); the other outcome was unknown.

SAEs were reported in 54 pediatric patients (Table S4), all with previous or ongoing medical conditions (Table S3). Of these 54 patients, 31 (57%) were aged 2 to <13 years, and 13 (24%) were aged 13 to <18 years. Fatal outcomes were recorded in 41 patients <18 years of age (Table 5), including five infants (aged ≤6 months). The most common SAEs reported in pediatric patients with a fatal outcome were respiratory failure (27%), acute respiratory distress syndrome (12%), and death (12%). The most common drug‐related SAEs reported were cholestasis (4%) and renal failure (4%) (Table S4).

## 4 DISCUSSION

This report summarizes characteristics and safety events of 4,033 patients from 38 countries treated with IV or nebulized zanamivir in a global CUP conducted between 2009 and 2019. Approximately half of these patients were based in the United Kingdom, and around 15% in the United States. The high uptake in the United Kingdom could be due to consistent support from Public Health England (PHE) for use of neuraminidase inhibitors in influenza.^18^ The PHE guidance has included IV zanamivir in its algorithm to guide selection of antiviral therapy since 2015, with details of the CUP until its closure.^19^ It was also observed that some physicians in the United Kingdom made repeat requests of IV zanamivir for additional patients.

The subpopulation presented in the CRF generally displayed more severe pre‐existing conditions and comorbidities and higher numbers of SAEs, compared with the overall CUP population. Since CRF completion was not mandated, it is possible that a more serious condition when hospitalized and/or a serious outcome may have prompted the treating physicians to complete the CRF, with the highest completion and return rate (over half of patients) observed in the United States.

Over 95% of requests in the MSTS were for IV zanamivir. The reason for the preference may be due to rapid and reliable systemic exposure of IV zanamivir to patients in an intensive care unit setting, including those with complicated influenza, compared with inhaled or oral antivirals.^20^ In addition, topical distribution to the lungs through inhalation may be obstructed by severe lower respiratory tract viral infection and/or bacterial superinfection with lung consolidation.^19^ Furthermore, pathogenic influenza strains may manifest as extrapulmonary disease where systemic antiviral treatment is advantageous.

Immunocompromised patients and young children have an increased likelihood to harbor viruses with reduced antiviral drug susceptibility.^21^, ^22^ This was observed in the CUP population, as a substantial number of patients had underlying respiratory illness and were immunocompromised. These factors could lead to an extended treatment duration per the Physician Guidance Document.^21^ A second treatment course beyond 5 days was requested for almost a quarter of patients, and a third or fourth course for almost 5%. A pharmacokinetic study of IV zanamivir in hospitalized neonates and infants with influenza infection^23^ and a pregnancy registry have been established to collect additional information from these high‐risk groups.^24^ IV zanamivir exposure in infants aged ≤6 months in the CUP, although limited, also supported the design of the post‐registration pharmacokinetic and safety study described above (ClinicalTrials.gov: NCT04494412).

No new safety concerns or signals were identified for IV zanamivir, and the overall SAE profile of IV zanamivir was similar to that reported in the clinical development program.^13^, ^14^, ^25^, ^26^ Based on data from the CRF database, the mortality rate during the CUP was approximately 26%. The GSK safety database contained records for 466 patients having experienced ≥1 SAE while receiving IV or nebulized zanamivir in the CUP; 374 had a fatal outcome, providing a mortality rate of 374/4,033 (~9%) patients. The higher mortality rate reported in CRF is probably driven by more serious conditions and outcomes in the CRF cohort than the overall CUP population. It was mandatory for physicians to report SAEs (including deaths), but optional to return CRFs; hence, mortality rates could be more accurately described using the GSK safety database. Nonetheless, the actual mortality rate in the CUP may have been higher than indicated by the GSK safety database: The CUP was not a clinical study, so there were neither monitoring visits nor source document verifications, which may have led to underreporting of events. Data from the CUP indicated no increase in SAEs or fatal SAEs in the pediatric population compared with the adult population, contrary to previous reports.^27^ Based on data from the GSK safety database, up to 24% of children experienced a SAE compared with 22% of adults. Mortality rates from the GSK safety database were similar in adults (9%) and children (10%) (Figure S2A,B).

Data presented here complement data from the IV zanamivir development program^8^, ^9^, ^28^ and phase II and III clinical trials.^13^, ^14^, ^25^ The open‐label, international, phase II trial in adult patients reported SAEs in 34% of patients and 14‐ and 28‐day all‐cause mortality rates of 13% and 17%.^13^ In the phase II, open‐label, multicenter study in children (≥6 months and <18 years of age), 32% experienced grade 3 or 4 AEs, and the mortality rate was 7%.^25^ The phase III trials reported SAEs in 16% and 19% of patients in the IV zanamivir 300‐ and 600‐mg groups, respectively, and all‐cause mortality rates of 7%.^14^ The CUP provides additional safety data about the compassionate use of IV zanamivir from a clinical setting and offers insight on the use of IV zanamivir in high‐risk patient populations who would normally be excluded from the registration studies. These include infants aged ≤6 months, pregnant women, and other individuals with severe comorbidities or complications when infected (e.g., immunocompromised patients).

The major strength of this analysis is the prospective data collection enabling a comprehensive evaluation of demography and safety data from an enriched patient population with hospitalized influenza in a real‐world setting. This program enabled direct and quicker access to emergency treatment with IV zanamivir prior to its marketing approval on a global scale, potentially saving lives of severely ill patients with influenza. As the CUP lasted a decade, it also encompassed the swine‐flu pandemic and influenza seasons of different severity with different circulating influenza strains. At the time of product registration, CUP data have also provided insight into the safety profile of IV zanamivir when used in high‐risk populations, including infants, pregnant women, and critically ill patients; such information would normally only become available through post‐marketing authorization surveillance.^13^, ^14^

The limitations of this analysis should be considered. As the CUP was not a clinical study, the quality and completeness of data cannot be assured. CRF completion rate was low (<25%) and may have been biased towards patients with a worse outcome. Additionally, as most CRFs were completed in the United Kingdom and the United States, some of the findings of this CUP may not represent the global population. It is also possible that outcomes such as mortality rates and SAEs were underreported.^27^, ^29^ No efficacy or virology data, other than partial clinical outcome and resistance data, were reported. Additionally, integration across the MSTS, CRF, and GSK safety databases was not feasible. Complete birth dates were not recorded for many patients in the CUP; this was according to privacy principles to collect only the data needed for the purpose of providing compassionate use medicine that came into effect during the CUP. This made identifying the age of children, particularly those <1 year of age, and cross‐referencing patients across databases difficult or impossible. Moreover, a causal relationship between zanamivir and SAEs is difficult to establish due to confounding symptoms of severe influenza, severe or chronic underlying disease, and overall critical illness. As the CUP was not a clinical study, there was a lack of an active comparator; therefore, an association between zanamivir and clinical outcome could not be fully assessed.

In conclusion, the CUP accomplished its goal of providing zanamivir aqueous solution to seriously ill, hospitalized patients with influenza infection globally, many of whom had no alternative treatment options for emergency use prior to product approval. The safety profile for zanamivir in the CUP was consistent with previous reports, and no new safety concerns were identified. The data collected have not changed the benefit–risk profile that has already been established for zanamivir.

## AUTHOR CONTRIBUTIONS

**Jie Wang‐Jairaj**: Writing – review & editing;  **Irene Miller:** Data curation; investigation; methodology and writing – review & editing. **Aditya Joshi:** Formal analysis; software and writing – review & editing. **Tharaka Jayabalan:** Data curation; investigation and writing – review & editing. **Amanda Peppercorn:** Conceptualization; funding acquisition; methodology; supervision and writing – review & editing. **Peter Zammit‐Tabona:** Data curation; investigation; methodology; resources and writing – review & editing. **Amanda Oliver:** Writing – review & editing.

## FUNDING INFORMATION

This analysis was funded by GlaxoSmithKline (GSK ID: 113375). Case report form processing and data management were provided by Parexel and PPD and were funded by GSK.

## CONFLICTS OF INTEREST

Aditya Joshi, Irene Miller, Tharaka Jayabalan, Peter Zammit‐Tabona, Amanda Peppercorn and Amanda Oliver are employees of and hold stocks/shares in GSK. Jie Wang‐Jairaj was a former employee of GSK at the time of the CUP and holds stock/shares in GSK.

## ETHICS APPROVAL STATEMENT

Ethics approvals were obtained in accordance with local regulations.

## PATIENT CONSENT STATEMENT

Before IV zanamivir administration, the treating physician obtained informed consent from the patient or legal guardian. A separate form was provided by GSK to obtain consent to share medical information; the latter was distinct from consent to treatment and was not a prerequisite for supply of zanamivir.

### PEER REVIEW

The peer review history for this article is available at https://publons.com/publon/10.1111/irv.12947.

## Supporting information


**Table S1:** Summary of modifications to data collected by the CRF
**Table S2:** Summary of information captured by MSTS, CRF and GSK Safety datasets^†^
**Table S3:** Summary of historical and current medical conditions (combined) by preferred term in patients with ≥1 SAE, reported for ≥1% of all patients or ≥1% of pediatric (<18 years of age) patients recorded on GSK Safety Database
**Table S4:** Drug‐related SAEs reported in ≥1% of all pediatric patients included in the GSK safety database
**Figure S1:** Overview of CUP process for requesting treatment and data processing after treatment is administered
**Figure S2:** Frequency and percentages of serious adverse event (SAE) cases reported by age group*

## Data Availability

The data that support the findings of this study are available in the supplementary material of this article.

## References

[1] World Health Organization . Influenza (seasonal) 2018; https://www.who.int/news-room/fact-sheets/detail/influenza-(seasonal). Accessed 15 June 2020.

[2] Bright RA , Medina MJ , Xu X , et al. Incidence of adamantane resistance among influenza A (H3N2) viruses isolated worldwide from 1994 to 2005: a cause for concern. Lancet (London, England). 2005;366(9492):1175‐1181. 10.1016/S0140-6736(05)67338-2 16198766

[3] McKimm‐Breschkin JL . Influenza neuraminidase inhibitors: antiviral action and mechanisms of resistance. Influenza Other Respir Viruses. 2013;7(Suppl 1):25‐36. 23279894 10.1111/irv.12047 PMC4942987

[4] O'Hanlon R , Shaw ML . Baloxavir marboxil: the new influenza drug on the market. Curr Opin Virol. 2019;35:14‐18. 30852344 10.1016/j.coviro.2019.01.006

[5] Shionogi Pharma Co . Full prescribing information of Xofluza (Baloxavir marboxil). 2019.

[6] European Centre for Disease Prevention and Control . Expert opinion on neuraminidase inhibitors for the prevention and treatment of influenza—review of recent systematic reviews and meta‐analyses. 2017.

[7] Shetty AK , Peek LA . Peramivir for the treatment of influenza. Expert Rev Anti Infect Ther. 2012;10(2):123‐143. 22339187 10.1586/eri.11.174

[8] Lackenby A , Besselaar TG , Daniels RS , et al. Global update on the susceptibility of human influenza viruses to neuraminidase inhibitors and status of novel antivirals, 2016‐2017. Antiviral Res. 2018;157:38‐46. 29981793 10.1016/j.antiviral.2018.07.001 PMC6094047

[9] Nguyen HT , Sheu TG , Mishin VP , Klimov AI , Gubareva LV . Assessment of pandemic and seasonal influenza A (H1N1) virus susceptibility to neuraminidase inhibitors in three enzyme activity inhibition assays. Antimicrob Agents Chemother. 2010;54(9):3671‐3677. 20585136 10.1128/AAC.00581-10 PMC2934949

[10] European Medicines Agency . Dectova. 2019; https://www.ema.europa.eu/en/medicines/human/EPAR/dectova. Accessed 25 June 2020.

[11] Oxford JS . Zanamivir (Glaxo Wellcome). IDrugs. 2000;3(4):447‐459. 16100701

[12] Watanabe A , Yates PJ , Murayama M , Soutome T , Furukawa H . Evaluation of safety and efficacy of intravenous zanamivir in the treatment of hospitalized Japanese patients with influenza: an open‐label, single‐arm study. Antivir Ther. 2015;20(4):415‐423. 25470818 10.3851/IMP2922

[13] Marty FM , Man CY , van der Horst C , et al. Safety and pharmacokinetics of intravenous zanamivir treatment in hospitalized adults with influenza: an open‐label, multicenter, single‐arm, phase II study. J Infect Dis. 2014;209(4):542‐550. 23983212 10.1093/infdis/jit467 PMC4047294

[14] Marty FM , Vidal‐Puigserver J , Clark C , et al. Intravenous zanamivir or oral oseltamivir for hospitalised patients with influenza: an international, randomised, double‐blind, double‐dummy, phase 3 trial. Lancet Respir Med. 2017;5(2):135‐146. 28094141 10.1016/S2213-2600(16)30435-0

[15] GlaxoSmithKline . Compassionate use (expanded access). https://www.gsk.com/en-gb/research-and-development/trials-in-people/compassionate-use-expanded-access

[16] GlaxoSmithKline . Zanamivir aqueous solution for compassionate use in serious influenza illness. GSK; 2019.

[17] Wang‐Jairaj J , Zammit‐Tabona P , Miller I , et al. Aqueous Zanamivir global Compassionate Use Program—2009‐2019. Paper presented at: Options X for the Control of Influenza. Abstract no 10978. 2019; Singapore.

[18] Public Health England . The use of antivirals for the treatment and prophylaxis of influenza. 2014; https://assets.publishing.service.gov.uk/government/uploads/system/uploads/attachment_data/file/777455/AV_full_guidance.pdf. Accessed 16 November 2014.

[19] Public Health England . PHE guidance on use of antiviral agents for the treatment and prophylaxis of seasonal influenza. 2019; https://www.gov.uk/government/publications/influenza-treatment-and-prophylaxis-using-anti-viral-agents. Accessed 14 September 2020.

[20] European Medicine Agency . Summary on compassionate use for IV Zanamivir 23 June 2011.

[21] Gubareva LV , Matrosovich MN , Brenner MK , Bethell RC , Webster RG . Evidence for zanamivir resistance in an immunocompromised child infected with influenza B virus. J Infect Dis. 1998;178(5):1257‐1262. 9780244 10.1086/314440

[22] Ison MG , Gubareva LV , Atmar RL , Treanor J , Hayden FG . Recovery of drug‐resistant influenza virus from immunocompromised patients: a case series. J Infect Dis. 2006;193(6):760‐764. 16479508 10.1086/500465

[23] GlaxoSmithKline . An intravenous (IV) zanamivir pharmacokinetics (PK) study in hospitalized neonates and infants with influenza infection, NCT04494412. 2021; https://clinicaltrials.gov/ct2/show/NCT04494412 Accessed 24 June 2020.

[24] UK Obstetric Surveillance System (UKOSS) . New therapies for influenza 2020; https://www.npeu.ox.ac.uk/ukoss/current-surveillance/therapiesflu. Accessed 24 June 2020.

[25] Bradley JS , Blumer JL , Romero JR , et al. Intravenous zanamivir in hospitalized patients with influenza. Pediatrics. 2017;140(5):e20162727. 29051331 10.1542/peds.2016-2727

[26] Electronic Medicines Compendium . DECTOVA SMpC 2019; https://www.medicines.org.uk/emc/product/10193/smpc. Accessed 15 June 2020.

[27] Sedani . Zanamivir aqueous solution for treatment of influenza in a compassionate use program: results from a retrospective chart review study, NAI115008. Paper presented at: Options for the Control of Influenza VIII. Abstract no: P1‐274. 2013; Cape Town, South Africa.

[28] European Medicines Agency . Dectova 2019; https://www.ema.europa.eu/en/medicines/human/EPAR/dectova

[29] Cleary PR , Crofts J , Parry‐Ford F , Chand M , Phin N . Characteristics and mortality of severe influenza cases treated with parenteral aqueous zanamivir, United Kingdom, October 2009 to January 2011. Influenza Other Respi Viruses. 2019;13(1):44‐53. 10.1111/irv.12603 PMC6304314 30137684

