# Supplementary material for: Zanamivir aqueous solution in severe influenza: A global Compassionate Use Program, 2009–2019
Source: Influenza Other Respir Viruses. 2021 Dec 22;16(3):542–51. doi: 10.1111/irv.12947 (PMC8983904; doi:10.1111/irv.12947)
Supplement: Supplementary file 1 — Table S1: Summary of modifications to data collected by the CRF Table S2: Summary of information captured by MSTS, CRF and GSK Safety datasets† Table S3: Summary of historical and current medical conditions (combined) by preferred term in patients with ≥1 SAE, reported for ≥1% of all patients or ≥1% of pediatric (<18 years of age) patients recorded on GSK Safety Database Table S4: Drug‐related SAEs reported in ≥1% of all pediatric patients included in the GSK safety database Figure S1: Overview of CUP process for requesting treatment and data processing after treatment is administered Figure S2: Frequency and percentages of serious adverse event (SAE) cases reported by age group* [file IRV-16-542-s001.docx]

# SUPPLEMENTARY METHODS

## CUP initiation and design

Zanamivir aqueous solution was dispensed to treating physicians following requests submitted through a clinical support help desk, which triaged calls to the program. Contact details were available from GSK locally, government sources or through word of mouth. All requests received for the program were treated with urgency, with a target of drug delivery to hospitals within 24 hours of initial contact. Following initial contact, the requesting physician was provided with the Physicians Guidance document (PGD), similar to a protocol, which was used to determine dosing of zanamivir based on age and renal function of the patient. In adolescents, children and infants with normal renal function, weight was also used to determine the correct dose. A request form was also provided, requesting completion of essential patient information and the number of vials of zanamivir required for a 5-day course of treatment. This information was recorded in the master summary tracking sheet (MSTS) and was required if the treating physician requested an additional course for a patient, in which case, a new request was submitted for an additional 5-day course of treatment.

The decision to treat was made by the physician and the request form included a declaration for physicians for this purpose. Prior to administration of IV zanamivir, the treating physician was responsible to obtain informed consent for treatment from the patient or their legal guardian. Documentation for consent was retained at sites. If required, ethics and regulatory approvals were also obtained, in accordance with local regulations. A separate informed consent form was provided by GSK, which was used for consent to share medical information; the latter was distinct from consent to treatment and was not a prerequisite for supply of zanamivir. It was also the requesting physician’s responsibility to follow local compassionate use regulatory processes, although where possible, GSK provided support and guidance to help expedite delivery. Wherever possible, delivery was made from local depots, to enable an expedited supply.

## Dosing and administration

Treatment beyond 5 days could be considered by the treating physician depending on the patient’s clinical status, including ongoing critical illness (e.g., respiratory failure, multi-organ failure, intensive care unit setting, and severe underlying immune suppression), continued viral shedding, or unresolved clinical influenza illness, and with reference to the PGD. Renal function or emergent renal insufficiency monitoring was recommended daily during treatment (serum creatinine and calculated creatinine clearance) to determine if dose modifications were required.

## Eligibility criteria

In addition to being hospitalized and being severely ill with influenza infection, the patient was eligible for treatment if approved anti-influenza drugs were ineffective, or for whom drug delivery by a route other than IV was not expected to be dependable or was not feasible, or the patient was infected with a documented influenza virus that was resistant to other approved antiviral agents.

Patients were not eligible for treatment in the CUP if they were pregnant (unless expected benefit to the patient was thought to outweigh any possible risk to the fetus), were known or suspected to be hypersensitive to zanamivir, or were receiving medical care at a facility participating in the Phase II pediatric/adult^1,2^ (NCT01014988) or Phase III adult^3^ (NCT01231620) clinical trials of IV zanamivir while they were ongoing and the patient was suitable for participation in these studies. Patients who had been enrolled in either clinical trial and were discontinued from the trial for reasons unrelated to patient safety, but who in the opinion of the treating physician might still derive benefit from IV zanamivir treatment, were eligible for treatment within the CUP.

## Data collection

Common identifiers included country, sex and age; no unique patient identifiers were provided across the databases. Data across the three data sources are reported separately due to incomplete data sets for some patients, including, but not limited to, date of birth. For this reason, it was not possible to calculate age for all patients, and data across the three data sources could not be merged. All patient information was anonymized.

## Adverse event reporting

Abnormal laboratory test results judged by the treating physician to be clinically significant were recorded as adverse events (AEs) (CRF only) or serious adverse events (SAEs), including hematology, clinical chemistry, urinalysis or other safety assessments (such as electrocardiograms, radiological scans, vital signs measurements), including those that worsened from baseline.

Any clinically significant safety assessments that were associated with the underlying disease, unless judged by the treating physician to be more severe than expected for the patient’s condition, were not reported as AEs or SAEs.

# Tables

## Supplementary Table 1: Summary of modifications to data collected by the CRF

| **CRF Version 8 (20 Jan 2012)** | **CRF Version 4 (16 Feb 2010)** | **CRF Version 3 (01 Oct 2009)** | **Data Collection Form (CRF version 2)** |
| --- | --- | --- | --- |
| Program identifier: REL113375  Patient Identifier (US Pts include EIND):  For GSK use only | Program identifier: REL113375  Patient Identifier: REL113375 -  Emergency IND Number (US only) | Program identifier: REL113375  Patient Identifier: REL113375 -  Emergency IND Number (US only) | Emergency IND Number (US only) |
| Name of treating physician  Address  Country | Name of Treating Physician  Address  Phone/FAX  Email  Country | Name of Treating Physician  Address  Phone/FAX  Email  Country | Treating Physician: Name,  Address  Phone/ FAX  Email |
| Treating physician signature and date  Treating physician’s name | Treating Physician’s signature and date  Treating physician’s name | Treating Physician’s signature and date  Treating physician’s name |  |
| **DEMOGRAPHY**  Date of birth *(ddmmmyy)**  Sex *(M=Male, F=Female)*  Weight *(kg)*  Ethnicity *(1=Hispanic or Latino, 2=Not Hispanic or Latino)*  Geographic Ancestry *(1=African American/African Heritage, 2=American Indian or Alaskan Native, 3= Asian-Central/South Asian Heritage, 4=Asian-East Asian Heritage, 5=Asian-Japanese Heritage, 6=Asian-South East Asian Heritage, 7=Native Hawaiian or other Pacific Islander, 8=White-Arabic/North African Heritage, 9=White-White/Caucasian/European Heritage)* | **DEMOGRAPHY**  Date of birth *(ddmmmyy)**  Sex *(M=Male, F=Female)* | **DEMOGRAPHY**  Date of birth *(ddmmmyy)**  Sex *(M=Male, F=Female)* | **Patient Information**:  Patient Number  Date of Birth *(ddmmmyy)**  Sex  Ethnicity  Country   1. **BASELINE INFORMATION/BRIEF Medical HISTORY:**   Please summarize baseline clinical status (including vital signs) and concurrent medical conditions (including underlying chronic disease or pregnancy) prior to zanamivir treatment. Please comment on the nature of influenza symptoms and the date of onset of symptoms.   1. **CONCURRENT INTERVENTIONS:**   Please record medications and any interventions (e.g. mechanical ventilation, supplemental oxygen) administered concurrently with zanamivir.  Date  Concurrent Medications/Interventions   1. **INFLUENZA DIAGNOSIS:**   Please record information on diagnosis of influenza infection.  Date of sample  Method (PCR, culture, rapid assay)  Result of test (+/-)  Influenza type/subtype   1. **VIROLOGY SAMPLES:**   If samples were collected for analysis by GlaxoSmithKline, please record details below. Please include information on source and timing of the sample.  Date of sample  Source/type of sample (e.g. nasopharyngeal swab)  Timing of sample (e.g. pre- during- or post-treatment)   1. **PHARMACOKINETIC SAMPLES:**   If samples were collected for analysis by GlaxoSmithKline, please record details below. Please include information on date/time of sample collection as well as date/time of the most proximal zanamivir dose in each case.  Date Sample Collected  Time Sample Collected  Date Dose Administrated  Time Dose Started  Time Dose Ended   1. **ZANAMIVIR TREATMENT:**   Please record mode of administration and dosing details  Check one: Intravenous, Inhaled Nebulized  Initial Treatment Course (Date, Dose/Frequency)  Additional Treatment Course (if applicable) (Date, Dose/Frequency)   1. **CLINICAL COURSE/OUTCOME:**   Please record details of clinical progression of disease and outcome. Please include information on relevant assessments and measurements (e.g. vital signs, influenza symptoms, oxygen saturation, chest X-rays) and length of hospitalization.   1. **SERIOUS ADVERSE EVENTS**   If the patient experienced a serious adverse event (SAE), please complete the separate SAE form provided and send the form to GSK per the instructions in the Physician Guidance Document. |
| **PREGNANCY INFORMATION**  Is the patient pregnant? *(Y=Yes, N=No, X=N/A (not of childbearing potential or male))*  If yes, check one *(1=First Trimester, 2=Second Trimester, 3=Third Trimester)* | **PREGNANCY INFORMATION**  Is the patient pregnant? *(Y=Yes, N=No, U=Not Applicable (not of childbearing potential or male))*  If yes, X one *(1=First trimester, 2=Second Trimester, 3=Third Trimester)* | **PREGNANCY INFORMATION**  Is the patient pregnant? *(Y=Yes, N=No, U=Not Applicable (not of childbearing potential or male))*  If yes, X one *(1=First trimester, 2=Second Trimester, 3=Third Trimester)* |  |
| **CHRONIC UNDERLYING ILLNESS (CHRONIC ILLNESS)**  **Respiratory** *(1=Tobacco use, 2= Asthma, 3=Chronic obstructive pulmonary disease, 4=Chronic lung disease, 5=Sarcoidosis, 6=Lung Cancer, 7=Chronic supplementary oxygen)*  **Cardiovascular** *(1=Coronary Artery Disease, 2=Cardiomyopathy,3=Congestive Heart Failure, 4=Arrythmia, 5=Pacemaker/Defibrillator)*  **Renal Disease** *(1=Chronic renal insufficiency, 2=End stage renal disease: Hemodialysis, 3=End stage renal disease: Peritoneal dialysis)*  **Gastrointestinal Disease** *(1=Malnutrition, 2=Crohns Disease/Inflammatory Disease, 3=Cirrhosis/Chronic liver disease, 4= Morbid Obesity (BMI≥ 40)*  **Rheumatology and Immunology Disease** *(1=HIV/AIDS, 2=Leukemia/Lymphoma, 3=Rheumatoid Arthritis, 4=Organ/Bone marrow transplantation, 5=Vasculitis, 6=Lupus, 7=Any immunocompromise (including use of immunosuppressive medications))*  **Oncology** *(1=Current cancer or cancer treatment within previous year)*  **Neurology** *(1=Seizure disorder, 2=Dementia, 3=Stroke/Cerebral Vascular Disease, 4=Parkinson’s disease, 5=Multiple sclerosis, 6=Nursing home resident)*  **Endocrine Disease** *(1=Diabetes Mellitus, 2=Adrenal disease)*  **Newborn Prematurity** *(1=Necrotizing enterocolitis, 2=Congestive heart disease, 3=Lung disease, 4=CNS disease, 5=Low birth weight)* | **CHRONIC UNDERLYING ILLNESS (CHRONIC ILLNESS)**  **Respiratory** *(1=Tobacco use, 2= Asthma, 3=Chronic obstructive pulmonary disease, 4=Chronic lung disease, 5=Sarcoidosis, 6=Lung Cancer, 7=Chronic supplementary oxygen)*  **Cardiovascular** *(1=Coronary Artery Disease, 2=Cardiomyopathy,3=Congestive Heart Failure, 4=Arrythmia, 5=Pacemaker/Defibrillator)*  **Renal Disease** *(1=Chronic renal insufficiency, 2=End stage renal disease: Hemodialysis, 3=End stage renal disease: Peritoneal dialysis)*  **Gastrointestinal Disease** *(1=Malnutrition, 2=Crohns Disease/Inflammatory Bowel Disease, 3=Cirrhosis/Chronic liver disease, 4= Morbid Obesity (BMI> 40)*  **Rheumatology and Immunology Disease** *(1=HIV/AIDS, 2=Leukemia/Lymphoma, 3=Rheumatoid Arthritis, 4=Organ/Bone marrow transplantation, 5=Vasculitis, 6=Lupus, 7=Any immunocompromise (including use of immunosuppressive medications))*  **Oncology** *(1=Current cancer or cancer treatment within previous year)*  **Neurology** *(1=Seizure disorder, 2=Dementia, 3=Stroke/Cerebral Vascular Disease, 4=Parkinson’s disease, 5=Multiple sclerosis, 6=Nursing home resident)*  **Endocrine Disease** *(1=Diabetes Mellitus, 2=Adrenal disease)*  **Newborn Prematurity** *(1=Necrotizing enterocolitis, 2=Congestive heart disease, 3=Lung disease, 4=CNS disease, 5=Low birth weight)* | **CHRONIC UNDERLYING ILLNESS (CHRONIC ILLNESS)**  **Respiratory** *(1=Tobacco use, 2= Asthma, 3=Chronic obstructive pulmonary disease, 4=Chronic lung disease, 5=Sarcoidosis, 6=Lung Cancer, 7=Chronic supplementary oxygen)*  **Cardiovascular** *(1=Coronary Artery Disease, 2=Cardiomyopathy,3=Congestive Heart Failure, 4=Arrythmia, 5=Pacemaker/Defibrillator)*  **Renal Disease** *(1=Chronic renal insufficiency, 2=End stage renal disease: Hemodialysis, 3=End stage renal disease: Peritoneal dialysis)*  **Gastrointestinal Disease** *(1=Malnutrition, 2=Crohns Disease/Inflammatory bowel Disease, 3=Cirrhosis/Chronic liver disease, 4= Morbid Obesity (BMI> 40)*  **Rheumatology and Immunology Disease** *(1=HIV/AIDS, 2=Leukemia/Lymphoma, 3=Rheumatoid Arthritis, 4=Organ/Bone marrow transplantation, 5=Vasculitis, 6=Lupus, 7=Any immunocompromise (including use of immunosuppressive medications))*  **Oncology** *(1=Current cancer or cancer treatment within previous year)*  **Neurology** *(1=Seizure disorder, 2=Dementia, 3=Stroke/Cerebral Vascular Disease, 4=Parkinson’s disease, 5=Multiple sclerosis, 6=Nursing home resident)*  **Endocrine Disease** *(1=Diabetes Mellitus, 2=Adrenal disease)*  **Newborn Prematurity** *(1=Necrotizing enterocolitis, 2=Congestive heart disease, 3=Lung disease, 4=CNS disease, 5=Low birth weight)* |  |
| **Ventilation summary**  1=Non-invasive: CPAP *(Number of days)*  2=Non-invasive: BiPAP *(Number of days)*  3=ECMO *(Number of days)*  4=Endotracheal mechanical ventilation *(Number of days)* | **Ventilation summary**  1=Non-invasive: CPAP *(Number of days)*  2=Non-invasive: BiPAP *(Number of days)*  3=ECMO *(Number of days)*  4=Endotracheal mechanical ventilation *(Number of days)* | **Ventilation summary**  1=Non-invasive: CPAP *(Number of days)*  2=Non-invasive: BiPAP *(Number of days)*  3=ECMO *(Number of days)*  4=Endotracheal mechanical ventilation *(Number of days)* |  |
| **INFLUENZA DETAILS**  Date of first influenza symptoms onset *(ddmmmyy)*  Date of hospital admission *(ddmmmyy)*  If applicable, date of ICU admission *(ddmmmyy)*  Laboratory confirmation of H1N1? *(Y=Yes, N=No)*  Laboratory confirmation of other influenza virus? *(Y=Yes, N=No)*  If Yes, record details:  Record information on diagnosis of influenza infection.  Symptoms of influenza, X all that apply  1=Cough  2=Sore throat  3=Fever  4=Headache  5=Nasal symptoms (rhinorrhea, congestion)  6=Myalgias  7=Fatigue/malaise  8=Anorexia  9=Vomiting  10=Dyspnea  11=Nausea  12=Diarrhea | **INFLUENZA DETAILS**  Date of first influenza symptoms onset *(ddmmmyy)*  Date of hospital admission *(ddmmmyy)*  If applicable, date of ICU admission *(ddmmmyy)*  Laboratory confirmation of H1N1? *(Y=Yes, N=No)*  Laboratory confirmation of other influenza virus? *(Y=Yes, N=No)*  If Yes, record details:  Record information on diagnosis of influenza infection.  Symptoms of influenza, X all that apply  1=Cough  2=Sore throat  3=Fever  4=Headache  5=Nasal symptoms (rhinorrhea, congestion)  6=Myalgias  7=Fatigue/malaise  8=Anorexia  9=Vomiting  10=Dyspnea  10=Nausea  10=Diarrhea | **INFLUENZA DETAILS**  Date of first influenza symptoms onset *(ddmmmyy)*  Laboratory confirmation of H1N1? *(Y=Yes, N=No)*  Laboratory confirmation of other influenza virus? *(Y=Yes, N=No)*  If Yes, record details:  Symptoms of influenza, X all that apply  1=Cough  2=Sore throat  3=Fever  4=Headache  5=Nasal symptoms (rhinorrhea, congestion)  6=Myalgias  7=Fatigue/malaise  8=Anorexia  9=Vomiting  10=Dyspnea  11=Nausea  12=Diarrhea |  |
| **ZANAMIVIR – TREATMENT**  Please indicate route of zanamivir aqueous solution administration.  Intravenous, Nebulized, or Both Intravenous and Nebulized  What was the duration of treatment? *(days)*  Was the scheduled dosing adjusted during treatment due to change in renal function? *(Y=Yes, N=No)*  Was the scheduled dosing interrupted during the treatment period *(Y=Yes, N=No)*  Was zanamivir stopped prematurely before the scheduled end of treatment period? *(Y=Yes, N=No)*  If yes, please indicate one primary reason why the zanamivir stopped:  1=Adverse event  7=Treating physician’s discretion  specify  8=Decision by patient or proxy  Specify | **ZANAMIVIR – INTRAVENOUS**  Day  Date *(ddmmmyy)*  Actual dose  Units *(mg)*  Frequency *(e.g., BID)* | **ZANAMIVIR – INTRAVENOUS**  Day  Date *(ddmmmyy)*  Actual dose  Units *(mg)*  Frequency *(e.g., BID)* |  |
|  | **ZANAMIVIR – INHALED NEBULISED (If applicable)**  Day  Date *(ddmmmyy)*  Actual dose  Units *(mg)*  Frequency *(e.g., BID)* | **ZANAMIVIR – INHALED NEBULISED (If applicable)**  Day  Date *(ddmmmyy)*  Actual dose  Units *(mg)*  Frequency *(e.g., BID)* |  |
| **ANTI-INFLUENZA THERAPY (INLCUDES VACCINES)**  Drug Name *(Trade name preferred)*  Ongoing Medication? *(Y=Yes, N=No)* | **ANTI-INFLUENZA THERAPY (INLCUDES VACCINES)**  Drug Name  Start Date *(ddmmmyy)*  Stop Date *(ddmmmyy)*  Ongoing Medication? *(Y=Yes, N=No)* |  |  |
| **See the 2^nd^ section of Zanamivir Treatment above** | **ZANAMIVIR DISCONTINUATION**  Was zanamivir stopped prematurely before the scheduled end of treatment period? *(Y=Yes, N=No)*  If yes, one primary reason zanamivir was stopped:  1=Adverse event  7=Treating physician’s discretion  specify  8=Decision by patient or proxy  Specify |  |  |
|  | **Concomitant Medications**  Drug Name  Start Date *(ddmmmyy)*  Stop Date *(ddmmmyy)*  Ongoing Medication? *(Y=Yes, N=No)* |  |  |
| **CLINICAL COURSE/OUTCOME**  Clinical progression of disease and outcome:  1=Recovered/Resolved (i.e. discharged from hospital)  Date of ICU Discharge *(ddmmmyy)*  Date of Hospital Discharge *(ddmmmyy)*  2=Not recovered/Not resolved *(i.e. remains in hospital and record details below)*  3=Deceased *(Please complete SAE form)*  Since the initiation of treatment with zanamivir solution, were there emerging signs of impairment of function of significant toxicities in the following areas?  Cardiovascular *(Y=Yes, N=No)*  Hepatic *(Y=Yes, N=No)*  Renal *(Y=Yes, N=No)*  Gastrointestinal *(Y=Yes, N=No)*  Neurology *(Y=Yes, N=No)*  Hematology *(Y=Yes, N=No)*  Record details on clinical course /outcome including information on emerging signs of impairment of function or of significant toxicities below: | **CLINICAL COURSE/OUTCOME**  Clinical progression of disease and outcome:  1=Recovered/Resolved (i.e. discharged from hospital)  Date of ICU Discharge *(ddmmmyy)*  Date of Hospital Discharge *(ddmmmyy)*  2=Not recovered/Not resolved *(i.e. remains in hospital and record details below)*  3= Deceased *(Please complete SAE form)*  Since the initiation of treatment with zanamivir solution, were there emerging signs of impairment of function or of significant toxicities in the following areas?  Cardiovascular *(Y=Yes, N=No)*  Hepatic *(Y=Yes, N=No)*  Renal *(Y=Yes, N=No)*  Gastrointestinal *(Y=Yes, N=No)*  Neurology *(Y=Yes, N=No)*  Hematology *(Y=Yes, N=No)*  Record details on clinical course /outcome including information on emerging signs of impairment of function or of significant toxicities below: | **CLINICAL COURSE/OUTCOME**  Clinical progression of disease and outcome:  1=Recovered/Resolved (i.e. discharged from hospital)  2=Not recovered/Not resolved *(i.e. remains in hospital and record details below)*  3= Deceased *(Please complete SAE form)*  Since the initiation of treatment with zanamivir solution, were there emerging signs of impairment of function or of significant toxicities in the following areas?  Cardiovascular *(Y=Yes, N=No)*  Hepatic *(Y=Yes, N=No)*  Renal *(Y=Yes, N=No)*  Gastrointestinal *(Y=Yes, N=No)*  Neurology *(Y=Yes, N=No)*  Hematology *(Y=Yes, N=No)* |  |
| **ADVERSE EVENTS (AE) AND SERIOUS ADVERSE EVENT (SAE**)  Did the patient experience a serious adverse event during the study? *(Y=Yes, N=No)*  If Yes, complete the separate SAE form provided and send to GSK per the instructions in the Physician Guidance Document.  Did the patient experience any non-serious adverse events? *(Y=Yes, N=No)*  If Yes, complete the AE section below. | **ADVERSE EVENTS (AE) AND SERIOUS ADVERSE EVENT (SAE)**  Did the patient experience a serious adverse event during the study? *(Y=Yes, N=No)*  f Yes, complete the separate SAE form provided and send to GSK per the instructions in the Physician Guidance Document.  Did the patient experience any non-serious adverse events? *(Y=Yes, N=No)*  If Yes, complete the AE section below. | **SERIOUS ADVERSE EVENT (SAE)**  Did the patient experience a serious adverse event during the study? *(Y=Yes, N=No)*  If yes, complete the separate SAE form provided and send to GSK per the instructions in the Physician Guidance Document |  |
| **NON-SERIOUS ADVERSE EVENT (AE)**  Event  Start Date *(ddmmmyy)*  Start Time *(hr:min)*  Outcome *(1=Recovered/Resolved, 2=Recovering/Resolving, 3=Not recovered/Not resolved, 4=Recovered/Resolved with sequelae)*  End Date *(ddmmmyy)*  End Time *(hr:min)*  Frequency *(1=Single episode, 2=Intermittent)*  Maximum Intensity *(1=Mild, 2=Moderate, 3=Severe, X=Not applicable)*  Action Taken with zanamivir as a Result of the Non-Serious AE *(1=Zanamivir withdrawn, 2=Dose reduced, 3=Dose increased, 4=Dose not changed, 5=Dose interrupted, X=Not applicable)*  Relationship to zanamivir *(Is there a reasonable possibility that the AE may have been caused by zanamivir? Y=Yes, N=No)* | **NON-SERIOUS ADVERSE EVENT**  Event  Start Date *(ddmmmyy)*  Start Time *(hr:min)*  Outcome *(1=Recovered/Resolved, 2=Recovering/Resolving, 3=Not recovered/Not resolved, 4=Recovered/Resolved with sequelae)*  End Date *(ddmmmyy)*  End Time *(hr:min)*  Frequency *(1=Single episode, 2=Intermittent)*  Maximum Intensity *(1=Mild, 2=Moderate, 3=Severe, X=Not applicable)*  Action Taken with zanamivir as a Result of the Non-Serious AE *(1=Zanamivir withdrawn, 2=Dose reduced, 3=Dose increased, 4=Dose not changed, 5=Dose interrupted, X=Not applicable)*  Relationship to zanamivir *(Is there a reasonable possibility that the AE may have been caused by zanamivir? Y=Yes, N=No)* |  |  |

*Date of birth was requested but not always provided and latterly redacted in line with privacy principles

## Supplementary Table 2: Summary of information captured by MSTS, CRF and GSK Safety datasets^†^

|  | **MSTS**  **(N=4033)** | **CRF**  **(N=879)** | **GSK Safety database**  **(N=466)** |
| --- | --- | --- | --- |
| Country | √ | √ |  |
| Centre number | √ |  |  |
| EIND (MSTS and CRF) or patient number (MSTS and CRF) or patient ID (ARGUS only)^‡^ | √ | √ | √ |
| Argus case ID |  |  | √ |
| Date of birth (ddmmyy)^§^ | √ | √ |  |
| Age (years) |  |  | √ |
| Sex | √ | √ | √ |
| Ethnicity |  | √ |  |
| Dose information | √ | √ |  |
| Maintenance dose information | √ | √ |  |
| Route of administration^¶^ | √ | √ |  |
| Pregnancy status | √ |  | √ |
| Chronic underlying illness |  | √ |  |
| Baseline clinical status |  | √ |  |
| Clinical outcomes |  | √ |  |
| AE system organ class |  |  | √ |
| AE preferred term |  |  | √ |
| AE level seriousness |  |  | √ |
| Country of AE occurrence |  |  | √ |
| Case outcome |  |  | √ |
| Start date/time |  |  | √ |
| Event onset date/time |  |  | √ |
| Event outcome Date/Time |  |  | √ |
| Fatality flag |  |  | √ |
| Concurrent medical interventions or medications (including date) |  | √ |  |
| Influenza diagnosis (date of sample, method of diagnosis, influenza type/subtype) |  | √ |  |
| Concurrent medical condition |  |  | √ |
| Historical medical condition |  |  | √ |

^†^No cross-checks were required or carried out among the 3 datasets. ^‡^No unique patient identifiers were provided across the databases. ^§^Date of birth was requested but not always provided and latterly redacted in line with privacy principles. ^¶^Included IV or nebulized zanamivir or both.

AE, adverse event; CRF, case report; EIND, emergency investigational new drug; MSTS, master summary tracking sheet.

**Supplementary Table 3: Summary of historical and current medical conditions (combined) by preferred term in patients with ≥1 SAE, reported for ≥1% of all patients or ≥1% of pediatric (<18 years of age) patients recorded on GSK Safety Database**

| **Condition (preferred term)** | **All patients, n (%)**  **n=466** | **Pediatric patients, n (%)**  **n=54** |
| --- | --- | --- |
| **Any condition** | 384 (82) | 44 (81) |
| Acute respiratory distress syndrome | 78 (17) | 9 (17) |
| Mechanical ventilation | 56 (12) | 7 (13) |
| H1N1 influenza | 54 (12) | 8 (15) |
| Pyrexia | 53 (11) | 5 (9) |
| Influenza | 50 (11) | 5 (9) |
| Obesity | 47 (10) | 3 (6) |
| Dyspnea | 47 (10) | 6 (11) |
| Respiratory failure | 41 (9) | 8 (15) |
| Tobacco user | 39 (8) | 0 |
| Cough | 37 (8) | 3 (6) |
| Diabetes mellitus | 33 (7) | 1 (2) |
| Chronic obstructive pulmonary disease | 31 (7) | 0 |
| Pneumonia | 29 (6) | 4 (7) |
| Multiple organ dysfunction syndrome | 28 (6) | 4 (7) |
| Hypertension | 28 (6) | 2 (4) |
| Tobacco abuse | 24 (5) | 0 |
| Chronic kidney disease | 23 (5) | 0 |
| Sepsis | 22 (5) | 3 (6) |
| Immunosuppression | 22 (5) | 2 (4) |
| Myalgia | 22 (5) | 0 |
| Fatigue | 21 (5) | 1 (2) |
| Lung assist device therapy | 20 (4) | 1 (2) |
| Lung disorder | 20 (4) | 3 (6) |
| Renal failure | 20 (4) | 1 (2) |
| Acute kidney injury | 19 (4) | 0 |
| Leukemia | 18 (4) | 0 |
| Asthma | 18 (4) | 2 (4) |
| Immunodeficiency | 17 (4) | 1 (2) |
| Coronary artery disease | 17 (4) | 0 |
| Arrhythmia | 15 (3) | 0 |
| Malaise | 15 (3) | 1 (2) |
| Plasma cell myeloma | 14 (3) | 0 |
| Bone marrow transplant | 14 (3) | 2 (4) |
| Septic shock | 14 (3) | 4 (7) |
| Acute lymphocytic leukemia | 12 (3) | 4 (7) |
| Neoplasm malignant | 11 (2) | 1 (2) |
| Hypoxia | 10 (2) | 1 (2) |
| Respiratory distress | 10 (2) | 4 (7) |
| Renal transplant | 10 (2) | 0 |
| Acute myeloid leukemia | 10 (2) | 2 (4) |
| Lymphoma | 10 (2) | 0 |
| Hepatic cirrhosis | 10 (2) | 1 (2) |
| Graft versus host disease | 9 (2) | 0 |
| Cardiac failure | 8 (2) | 2 (4) |
| Lung transplant | 8 (2) | 0 |
| Stem cell transplant | 8 (2) | 0 |
| Cardiomyopathy | 8 (2) | 1 (2) |
| Alcoholism | 8 (2) | 0 |
| Diarrhea | 8 (2) | 0 |
| Seizure | 8 (2) | 2 (4) |
| Thrombocytopenia | 8 (2) | 1 (2) |
| Tobacco poisoning | 8 (2) | 0 |
| Vomiting | 7 (2) | 1 (2) |
| Cardiac failure congestive | 7 (2) | 0 |
| Pulmonary embolism | 7 (2) | 0 |
| Chemotherapy | 7 (2) | 2 (4) |
| Continuous positive airway pressure | 7 (2) | 0 |
| Malnutrition | 7 (2) | 0 |
| Deep vein thrombosis | 7 (2) | 1 (2) |
| Shock | 7 (2) | 1 (2) |
| Chronic lymphocytic leukemia | 6 (1) | 0 |
| Non-Hodgkin’s lymphoma | 6 (1) | 1 (2) |
| Type 2 diabetes mellitus | 6 (1) | 0 |
| Acute myocardial infarction | 6 (1) | 0 |
| Hospitalization | 6 (1) | 1 (2) |
| Acute respiratory failure | 6 (1) | 0 |
| Interstitial lung disease | 6 (1) | 1 (2) |
| Nasal disorder | 6 (1) | 0 |
| Pneumothorax | 6 (1) | 1 (2) |
| Hypothyroidism | 5 (1) | 0 |
| Renal impairment | 5 (1) | 0 |
| HIV infection | 5 (1) | 0 |
| Lung infiltration | 5 (1) | 0 |
| Allogenic stem cell transplantation | 5 (1) | 1 (2) |
| Endotracheal intubation | 5 (1) | 1 (2) |
| Hemodialysis | 5 (1) | 0 |
| Cerebrovascular accident | 5 (1) | 0 |
| Alcohol use | 5 (1) | 0 |
| Liver disorder | 5 (1) | 1 (2) |
| Gastrointestinal hemorrhage | 4 (<1) | 1 (2) |
| Cytomegalovirus infection | 4 (<1) | 1 (2) |
| Staphylococcal sepsis | 4 (<1) | 1 (2) |
| Cardiac arrest | 4 (<1) | 2 (4) |
| Extracorporeal circulation | 4 (<1) | 2 (4) |
| Pulmonary fibrosis | 4 (<1) | 1 (2) |
| Liver transplant | 3 (<1) | 2 (4) |
| Pulmonary hypertension | 3 (<1) | 1 (2) |
| Respiratory disorder | 3 (<1) | 1 (2) |
| Chest tube insertion | 3 (<1) | 1 (2) |
| Pneumonia bacterial | 3 (<1) | 1 (2) |
| Pneumonia staphylococcal | 3 (<1) | 1 (2) |
| Staphylococcal infection | 3 (<1) | 1 (2) |
| General physical health deterioration | 3 (<1) | 1 (2) |
| Vasculitis | 3 (<1) | 1 (2) |
| Premature baby | 3 (<1) | 3 (6) |
| Colitis ulcerative | 3 (<1) | 1 (2) |
| Bone marrow failure | 3 (<1) | 1 (2) |
| Bronchoalveolar lavage | 3 (<1) | 1 (2) |
| Hemodynamic instability | 2 (<1) | 1 (2) |
| Bronchitis | 2 (<1) | 1 (2) |
| Viral myocarditis | 2 (<1) | 2 (4) |
| Parenteral nutrition | 2 (<1) | 1 (2) |
| Acute leukemia | 2 (<1) | 1 (2) |
| Hypogammaglobulinemia | 2 (<1) | 1 (2) |
| Immune system disorder | 2 (<1) | 1 (2) |
| Asthenia | 2 (<1) | 1 (2) |
| Necrotizing colitis | 2 (<1) | 2 (4) |
| Epilepsy | 2 (<1) | 1 (2) |
| Febrile neutropenia | 2 (<1) | 1 (2) |
| Leukopenia | 2 (<1) | 1 (2) |
| Heart disease congenital | 2 (<1) | 2 (4) |
| Trisomy 21 | 2 (<1) | 1 (2) |
| Stenosis | 1 (<1) | 1 (2) |
| Adenovirus infection | 1 (<1) | 1 (2) |
| Bacterial sepsis | 1 (<1) | 1 (2) |
| Bronchiolitis | 1 (<1) | 1 (2) |
| Clostridium difficile colitis | 1 (<1) | 1 (2) |
| Cytomegalovirus viremia | 1 (<1) | 1 (2) |
| Device related sepsis | 1 (<1) | 1 (2) |
| Fungal infection | 1 (<1) | 1 (2) |
| Infection | 1 (<1) | 1 (2) |
| Pneumocystis jirovecii pneumonia | 1 (<1) | 1 (2) |
| Rhinovirus infection | 1 (<1) | 1 (2) |
| Cardiopulmonary bypass | 1 (<1) | 1 (2) |
| Chemotherapy multiple agents systemic | 1 (<1) | 1 (2) |
| Atelectasis | 1 (<1) | 1 (2) |
| Chronic respiratory failure | 1 (<1) | 1 (2) |
| Neonatal respiratory distress syndrome | 1 (<1) | 1 (2) |
| Pneumomediastinum | 1 (<1) | 1 (2) |
| Pulmonary edema | 1 (<1) | 1 (2) |
| Patent ductus arteriosus repair | 1 (<1) | 1 (2) |
| Thrombectomy | 1 (<1) | 1 (2) |
| Cardiopulmonary failure | 1 (<1) | 1 (2) |
| Acute lymphocytic leukemia recurrent | 1 (<1) | 1 (2) |
| Neuroblastoma | 1 (<1) | 1 (2) |
| Retinoblastoma | 1 (<1) | 1 (2) |
| Rhabdomyosarcoma | 1 (<1) | 1 (2) |
| Hypervolemia | 1 (<1) | 1 (2) |
| ABO incompatibility | 1 (<1) | 1 (2) |
| Anaphylactic reaction | 1 (<1) | 1 (2) |
| Heart transplant rejection | 1 (<1) | 1 (2) |
| Ileus | 1 (<1) | 1 (2) |
| Encephalomalacia | 1 (<1) | 1 (2) |
| Encephalopathy | 1 (<1) | 1 (2) |
| Jaundice | 1 (<1) | 1 (2) |
| Portal vein thrombosis | 1 (<1) | 1 (2) |
| Blood pressure increased | 1 (<1) | 1 (2) |
| Investigation | 1 (<1) | 1 (2) |
| Viral mutation identified | 1 (<1) | 1 (2) |
| Traumatic hemothorax | 1 (<1) | 1 (2) |
| Cerebral palsy | 1 (<1) | 1 (2) |
| Congenital absence of bile ducts | 1 (<1) | 1 (2) |
| Congenital musculoskeletal anomaly | 1 (<1) | 1 (2) |
| Dilatation intrahepatic duct congenital | 1 (<1) | 1 (2) |
| Duodenal atresia | 1 (<1) | 1 (2) |
| Intestinal atresia | 1 (<1) | 1 (2) |
| Mucopolysaccharidosis | 1 (<1) | 1 (2) |
| Fetal growth restriction | 1 (<1) | 1 (2) |

**Supplementary Table 4: Drug-related SAEs reported in ≥1% of all pediatric patients included in the GSK safety database**

| **Drug-related SAE (preferred term)** | **Paediatric patients, n (%) N=54** |
| --- | --- |
| Any drug-related SAE  Cholestasis | 13 (24)  2 (4) |
| Renal failure | 2 (4) |
| Hepatocellular injury | 1 (2) |
| Liver disorder | 1 (2) |
| Haemorrhage intracranial | 1 (2) |
| Seizure | 1 (2) |
| Renal injury | 1 (2) |
| Sinus bradycardia | 1 (2) |
| Alanine aminotransferase increased | 1 (2) |
| Aspartate aminotransferase increased | 1 (2) |
| Hepatic enzyme increased | 1 (2) |
| Oxygen saturation decreased | 1 (2) |
| Transaminases increased | 1 (2) |
| Intravascular haemolysis | 1 (2) |
| Vomiting | 1 (2) |
| Pyrexia | 1 (2) |
| Lower respiratory tract infection | 1 (2) |
| Toxicity to various agents | 1 (2) |
| Pulmonary haemorrhage | 1 (2) |
| Hypotension | 1 (2) |

SAE, serious adverse event

# Supplementary Figures

**Supplementary Figure 1: Overview of CUP process for requesting treatment and data processing after treatment is administered**


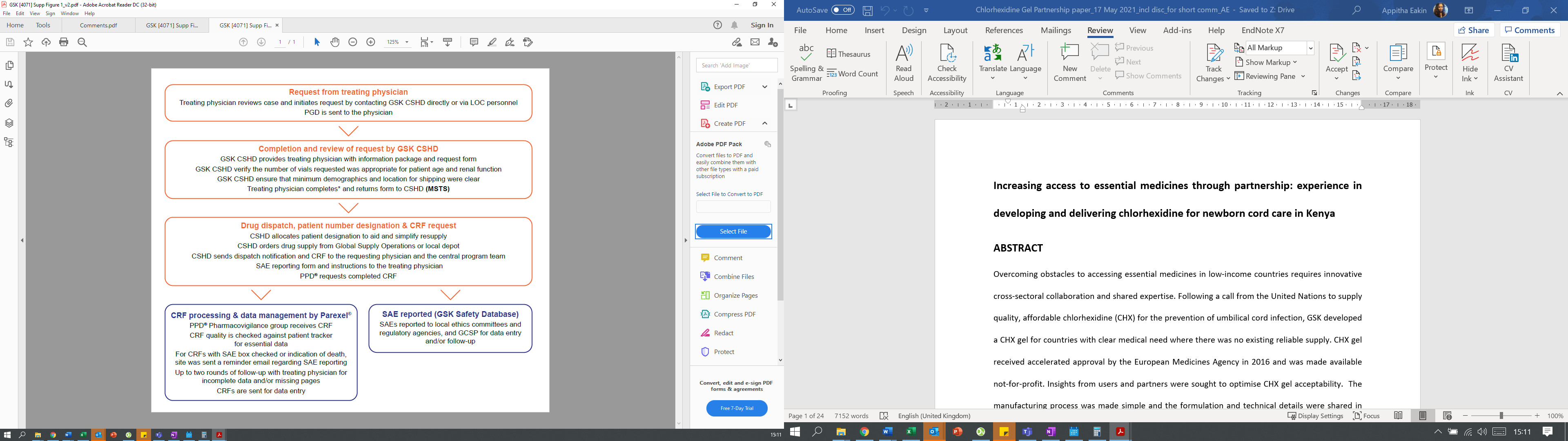


*Completion of request form carried out with support from the PPD Pharmacovigilance group, as required.

CRF, case report form; CSHD, Clinical Support Help Desk; GCSP, Global Clinical Safety and Pharmacovigilance, GSK, GlaxoSmithKline; LOC, Local Operating Company; MSTS, master summary tracking sheet; PGD, Physician Guidance Document; SAE, serious adverse event.

## Supplementary Figure 2: Frequency and percentages of serious adverse event (SAE) cases reported by age group*

**
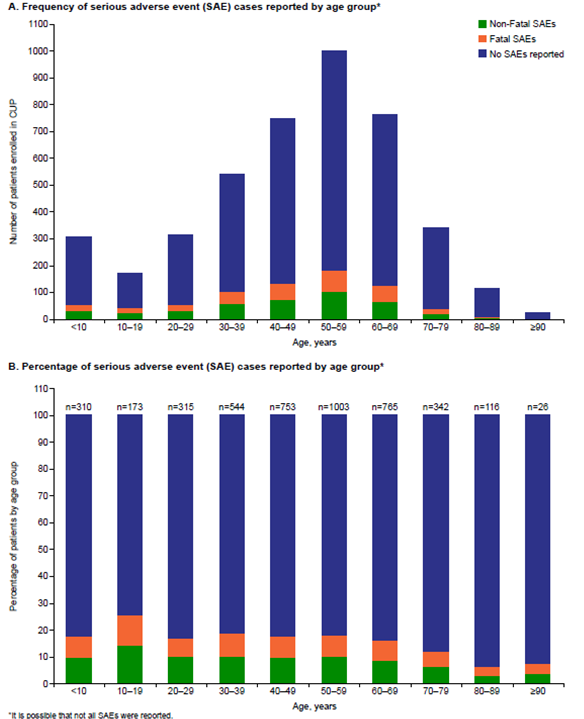
**

## References

1. Bradley JS, Blumer JL, Romero JR, et al. Intravenous Zanamivir in Hospitalized Patients With Influenza. *Pediatrics* 2017; **140**(5).

2. Marty FM, Man CY, van der Horst C, et al. Safety and pharmacokinetics of intravenous zanamivir treatment in hospitalized adults with influenza: an open-label, multicenter, single-arm, phase II study. *J Infect Dis* 2014; **209**(4): 542-50.

3. Marty FM, Vidal-Puigserver J, Clark C, et al. Intravenous zanamivir or oral oseltamivir for hospitalised patients with influenza: an international, randomised, double-blind, double-dummy, phase 3 trial. *Lancet Respir Med* 2017; **5**(2): 135-46.
